# Efficacy and prognosis biomarker of locally advanced ESCC patients treated with neoadjuvant chemotherapy and anti-PD-1 immunotherapy

**DOI:** 10.3389/fonc.2024.1498675

**Published:** 2025-02-19

**Authors:** Shuman Li, Jie Zhou, Qianli Wang, Jiewei Chen, Yapeng Qi

**Affiliations:** ^1^ Department of Medical Oncology, The Affiliated Cancer Hospital of Zhengzhou University & Henan Cancer Hospital, Zhengzhou, China; ^2^ Guangxi International Travel Healthcare Centre (Port Clinic of Nanning Customs District), Nanning, China; ^3^ Department of Pathology, Sun Yat-Sen University Cancer Center, Guangzhou, China; ^4^ Department of General Surgery, The Affiliated Cancer Hospital of Zhengzhou University & Henan Cancer Hospital, Zhengzhou, China

**Keywords:** esophageal squamous cell carcinoma, TAP, neoadjuvant therapy, prognosis biomarker, anti-PD-1 immunotherapy

## Abstract

**Introduction:**

Immunotherapy has rapidly advanced in tumor treatment. In esophageal squamous cell carcinoma (ESCC), its use in neoadjuvant therapy has shown promising results. Several phase III clinical trials have confirmed that immunodetection site inhibitors in neoadjuvant therapy can enhance the pathologically complete response (pCR) rate.

**Methods:**

We retrospectively analyzed 128 ESCC patients treated with neoadjuvant chemotherapy plus anti-PD-1 immunotherapy at the Affiliated Cancer Hospital of Zhengzhou University and Henan Cancer Hospital from July 2019 to November 2023.

**Results:**

Of the 128 patients, 31 (24.1%) achieved pCR, and 46 (35.9%) achieved a major pathological response (MPR). Female patients, low-level tumor abnormal protein (TAP), and moderate differentiation were significantly associated with a higher pCR rate and MPR rate. Besides pCR rate and MPR rate, low-level TAP and moderate differentiation had significantly longer PFS and OS. The mean PFS in the low-level TAP group was 42.4 months, significantly longer than the 28.5 months in the high-level TAP group (*p* = 0.019). The mean OS in the low-level TAP group was 43.7 months, compared to 30.5 months in the high-level TAP group (*p* = 0.027). The multivariate analysis showed that TAP and differentiation were independent prognostic factors for PFS, and the pCR rate was an independent prognostic factor for OS in ESCC patients treated with anti-PD-1. Thus, lower TAP levels predict a better response to neoadjuvant chemotherapy plus anti-PD-1 immunotherapy in advanced ESCC patients. In clinical practice, serum TAP levels before neoadjuvant therapy can serve as a useful tool to predict the efficacy of this combined treatment.

## Introduction

Esophageal cancer ranks as the seventh most common malignancy worldwide (1), comprising two main types: esophageal squamous cell carcinoma (ESCC) and esophageal adenocarcinoma. Esophageal adenocarcinoma mainly occurs in western developed countries. Esophageal adenocarcinoma is predominantly seen in Western developed countries, while ESCC is more common in China and African countries, with China accounting for over 90% of ESCC cases, particularly in the Taihang Mountains. Early-stage ESCC is typically treated with radical resection, whereas locally advanced cases are managed with neoadjuvant therapy, including radiotherapy and chemotherapy (2, 3). The CROSS and 5,010 studies have established neoadjuvant chemoradiotherapy (nCRT) as the standard treatment for locally advanced ESCC, achieving a pathologically complete response (pCR) in 29% of patients in the CROSS study (4).

Recent progress in immunotherapy, especially with immune checkpoint inhibitors (ICIs), has revolutionized tumor treatment. Clinical trials such as KEYNOTE-590, CheckMate 648, and ESCORT-1st have shown the effectiveness of combining chemotherapy and immunotherapy as a first-line treatment for late-stage ESCC. In China, most clinical studies have focused on neoadjuvant immunotherapy combined with chemotherapy due to the higher morbidity and mortality associated with neoadjuvant chemoradiotherapy (5). The cumulative toxic side effects, poor patient compliance, and increased surgical difficulty and complications have restricted the widespread use of chemoradiotherapy. Nonetheless, the NCT02844075 and PALACE-1 studies reported pCR rates of 56% and 46.1%, respectively, for neoadjuvant chemoradiotherapy combined with immunotherapy in locally advanced ESCC (6, 7). Additionally, the NICE study found a pCR rate of 45.4% with camrelizumab plus albumin-bound paclitaxel and carboplatin for locally advanced ESCC (8).

Many trials have demonstrated that while immunotherapy is effective as a neoadjuvant treatment for ESCC, a substantial number of patients do not benefit. This inefficacy leads to wasted medical resources and adverse reactions, including immune dermatitis, immunological hepatitis, and immune pneumonia (9). Thus, there is an urgent need to develop new biomarkers with high specificity and sensitivity to predict the efficacy of immunotherapy in neoadjuvant settings. Currently, tumor mutation burden (TMB), microsatellite instability, and PD-L1 protein expression are considered potential biomarkers (10). However, the effectiveness of PD-L1 expression as a predictor of patient response in ESCC is controversial. Studies such as KEYNOTE-590 (11)/Checkmate-648 (12)/JUPITER-06 (13) have shown that immunotherapy provides significant clinical benefits for locally advanced ESCC, regardless of PD-L1 status. In contrast, the NICE-1 study found no significant correlation between PD-L1 expression and pCR when combining neoadjuvant immunotherapy with chemotherapy (14). These studies highlight the need for further research to identify reliable biomarkers to predict immunotherapy response in ESCC patients.

Glycosylation is crucial in tumorigenesis (15), promoting tumor growth and metastasis (16). It also significantly impacts the immune system and signal transduction pathways, further contributing to tumor development (17). During glycosylation, various glycoproteins with abnormal glycan structures are produced on the cell surface (18). These aberrant glycoproteins can serve as vital biomarkers for cancer progression (19, 20).

The detection of abnormal glycosylated proteins serves as a reliable indicator of cancer progression (21, 22). The measurement of tumor abnormal proteins (TAP) relies on specific agglutinins that facilitate the aggregation of various glycoproteins, leading to the formation of distinctive crystalline condensates. These condensates, observable under a microscope, differ significantly from the non-specific debris typically found in serum. The areas of TAP crystalline condensates in blood samples can be employed for early cancer detection, accurate diagnosis, prognostic stratification, and monitoring the efficacy of treatment (21, 23, 24). During TAP analysis, these crystalline condensates, primarily composed of abnormal glycoproteins, are readily identifiable. However, the utility of TAP as a biomarker for ESCC and its correlation with the efficacy of neoadjuvant chemotherapy combined with anti-PD-1 immunotherapy remain unclear.

To address these questions, we retrospectively analyzed patients with locally advanced ESCC treated with neoadjuvant chemotherapy and anti-PD-1 antibodies. We assessed pathological response, tumor regression grade, TAP levels, clinical parameters, and survival outcomes.

## Patients and methods

### Patients

This retrospective study aimed to evaluate survival outcomes in patients with locally advanced ESCC who received neoadjuvant anti-PD-1 immunotherapy combined with chemotherapy at the Affiliated Cancer Hospital of Zhengzhou University and Henan Cancer Hospital. Data were collected from ESCC patients treated with neoadjuvant chemotherapy and anti-PD-1 antibodies from March 2020 to October 2023. The study was approved by our hospital’s Ethics Committee and adhered to the 2013 Declaration of Helsinki, with waived written informed consent. Eligible patients had ESCC classified as cT1N1-3M0 or cT2-4aN0-3M0 (AJCC, 8th edition), diagnosed via contrast-enhanced CT and/or upper gastrointestinal endoscopic ultrasonography (EUS). The inclusion criteria required the patients to be 18 years or older, with an Eastern Cooperative Oncology Group (ECOG) performance status (PS) of 0 or 1. The exclusion criteria included one case of secondary thyroid cancer, one case with severe bone marrow toxicity necessitating granulocyte colony-stimulating factor after initial treatment, and three cases receiving preoperative anti-PD1 combined with neoadjuvant radiochemotherapy. The baseline data collected included age, sex, body mass index (BMI), Eastern Cooperative Oncology Group Performance Status (ECOG-PS), tumor site, tumor differentiation, pathological response in resected specimens, and tumor, node, metastasis (TNM) staging.

### Neoadjuvant therapy and surgical procedures

The chemotherapy regimen comprised a taxane (albumin-bound paclitaxel, paclitaxel, or docetaxel) paired with a platinum compound (cisplatin, nedaplatin, or carboplatin), alongside anti-PD-1 immunotherapy options including camrelizumab, sintilimab, pembrolizumab, tislelizumab, or toripalimab. Surgical intervention was determined at the discretion of the surgeon following completion of at least two cycles of neoadjuvant therapy. The patients underwent contrast-enhanced CT re-evaluation within 1 week before surgery.

### TAP measurement

**Figure 1:**
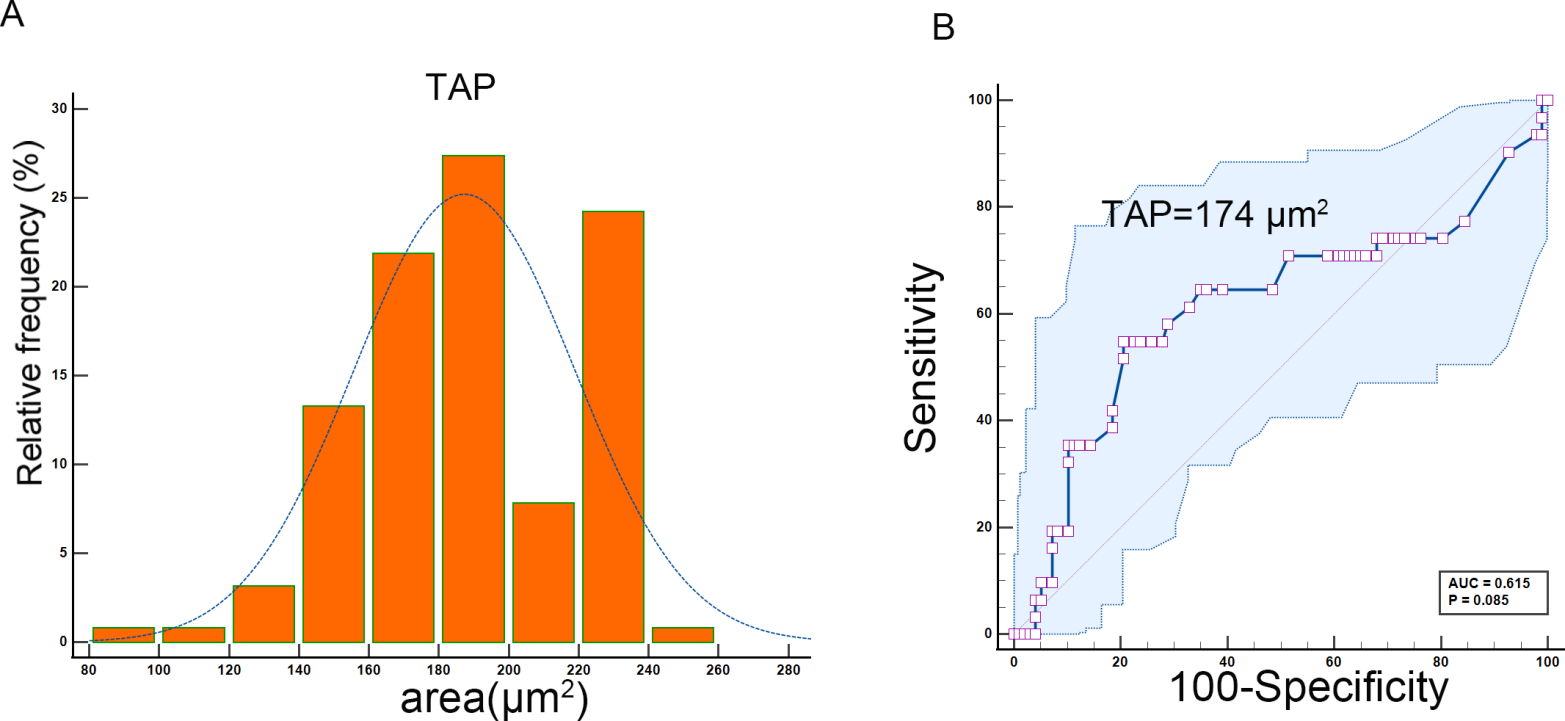
**(A)** Distribution of TAP. **(B)** The ROC curve determines the cutoff value of TAP.

Timing of sample collection: Samples were collected 1–7 days prior to neoadjuvant therapy or surgery. The TAP detection method, widely adopted in Chinese hospitals, involved the use of an abnormal glycan glycoprotein detection kit (agglutination-based), a TAP detection kit, and an image analyzer (Zhejiang Ruishen Medical Technology, Ltd., China). Fresh peripheral blood (25 µL) was collected via fingertip puncture and smeared to cover over two-thirds of a microscope slide. The slides were air-dried at room temperature, and three drops of the thoroughly mixed detection reagent were applied. After 1.5–2 h of natural drying, condensed particles indicative of TAP status were formed and analyzed using the TAP detection image analyzer. For further analysis, TAP levels were divided into two groups based on ROC curve analysis (**Figure 1**): high TAP group—particle area ≥174 µm²; low TAP group—particle area <174 µm².

### Assessment

Following neoadjuvant therapy, the pathological evaluation employed the College of American Pathologists (CAP)/National Comprehensive Cancer Network (NCCN) criteria. Responses were categorized into four levels: 0 (complete response), 1 (moderate response), 2 (mild response), and 3 (no response). Two pathologists (JQW and ZC) conducted a microscopic evaluation of all hematoxylin and eosin (HE) slides from our study’s enrolled patients.

### Statistical analysis

Statistical analyses were conducted using Medcalc, GraphPad Prism 9, and SPSS software (version 20.0; SPSS, Chicago, USA). Disease-free survival (DFS) and overall survival (OS) were estimated using Kaplan–Meier methods, with group differences assessed by using the log-rank test at a significance level of *p <*0.05. Hazard ratios (HRs) and their corresponding 95% confidence intervals (CIs) were calculated using the Cox proportional hazards model. The receiver operating characteristic (ROC) curve analysis determined the cutoff values. Pearson correlation analysis was employed to evaluate the association between TAP and baseline characteristics.

## Results

### Baseline characteristics

During the study, 128 patients with locally advanced ESCC underwent neoadjuvant chemotherapy and anti-PD-1 immunotherapy followed by surgery at the Affiliated Cancer Hospital of Zhengzhou University and Henan Cancer Hospital. The patient cohort included 96 male subjects (75%) with a median age of 64 years (range: 42–79 years). The tumors were primarily located in the middle (60 patients, 46.87%) and lower esophagus (52 patients, 40.63%). Most patients (110, 85.94%) had T3–4 stage tumors, and 59 patients (46.09%) had N2–3 stage lymph node involvement. The tumors were less than 2.9 cm in 70 patients (54.69%). Pathologically, 84 patients (65.62%) had moderately differentiated squamous cell carcinoma, while 26 patients (20.31%) had poorly differentiated squamous cell carcinoma. Detailed data are shown in **Table 1**. The correlation analysis showed that tumor size positively correlated with TAP (*p* = 0.037). Besides that, ESCC patients with tumor regression grade 0 tended to have a lower TAP. Among 31 patients with 0 grade, 17 (54.8%) patients had TAP lower than 174 µm² (*p* = 0.012). Detailed data are shown in **Table 2**.

**Table 1:** Patients’ characteristics.

Characteristics	Case
Age (year)	
≤64^a^	60 (46.88%)
>64	68 (53.12%)
Gender	
Male	96 (75%)
Female	32 (25%)
Tumor size (cm)	
≤2.9	70 (54.69%)
>2.9	58 (45.31%)
T stage	
T1–2	18 (14.06%)
T3–4	110 (85.94%)
N stage	
N0–1	49 (38.28%)
N2–3	59 (61.72%)
Tumor location	
Upper	16 (12.50%)
Middle	60 (46.87%)
Lower	52 (40.63%)
Differentiation	
Poor	26 (20.31%)
Well	18 (14.06%)
Moderate	84 (65.62%)

a Mean age.

**Table 2:** Patients’ characteristics and TAP.

	Case	TAP < 174 (µm²)	TAP ≥ 174 (µm²)	*p* ^a^
Age (year)				0.633
≤64	60	20 (33.3%)	40 (66.7%)	
>64	68	20 (29.4%)	48 (70.6%)	
Gender				1
Male	96	30 (31.3%)	66 (68.8%)	
Female	32	10 (31.3%)	22 (68.8%)	
Tumor size (cm)				**0.037**
≤2.9	70	27 (38.57%)	43 (61.42%)	
>2.9	58	13 (22.4%)	45 (77.6%)	
T stage				0.373
T1–2	18	4 (22.2%)	14 (77.8%)	
T3–4	110	36 (32.7%)	74 (67.3%)	
N stage				
N0–1	49	24 (34.8%)	25 (65.2%)	0.351
N2–3	59	16 (27.1%)	43 (72.9%)	
Tumor regression				**0.012**
0	31	17 (54.8%)	14 (45.2%)	
1	15	4 (26.6%)	11 (73.3%)	
2	65	16 (24.6%)	49 (75.4%)	
3	17	3 (17.6%)	14 (82.4%)	
Tumor location				0.346
Upper	16	3 (18.8%)	13 (81.3%)	
Middle	60	22 (36.7%)	38 (63.7%)	
Lower	52	15 (28.8%)	37 (71.2%)	
Differentiation				0.332
Poor	26	5 (19.2%)	21 (80.8%)	
Well	18	6 (33.3%)	12 (66.7%)	
Moderate	84	29 (34.5%)	55 (65.5%)	

a Chi-square test.

### Surgical and outcome data

Among the 128 patients who underwent surgery, all achieved R0 resection. Of these, 31 patients (24.22%) achieved a pCR, while 46 patients (35.94%) exhibited a major pathological response (MPR). Detailed surgical outcomes are shown in **Tables 3** and **4**. Female patients exhibited better pathological responses than male patients. Specifically, pCR was achieved in 40.6% of female subjects (13 patients) compared to 18.8% of male subjects (18 patients) (*p* = 0.012). Similarly, 53.1% of female subjects (17 patients) achieved MPR versus 30.2% of male subjects (29 patients) (*p* = 0.019). Tumor differentiation was also significantly correlated with pathological response. Moderately differentiated tumors had higher pCR (35.7%, *p* < 0.01) and MPR rates (52.4%, *p* < 0.01). Additionally, the level of TAP significantly influenced the pathological response. Among patients with ESCC and low TAP levels, 42.5% (17 patients) achieved pCR (*p* = 0.01), and 52.5% (21 patients) achieved MPR (*p* = 0.08).

**Table 3:** Characteristics and pCR.

	Case	pCR	No pCR	*p* ^a^
Age (year)				0.846
≤64	60	15 (25.0%)	45 (75.0%)	
>64	68	16 (23.5%)	52 (76.5)	
Gender				**0.012**
Male	96	18 (18.8%)	78 (81.3%)	
Female	32	13 (40.6%)	19 (59.4%)	
Tumor size (cm)				0.664
≤2.9	70	18 (25.7%)	52 (74.3%)	
>2.9	58	13 (22.4%)	45 (77.6%)	
T stage				0.117
T1–2	18	11 (38.9%)	7 (61.1%)	
T3–4	110	24 (21.8%)	86 (78.2%)	
N stage				0.769
N0–1	49	16 (23.2%)	53 (76.8%)	
N2–3	59	15 (25.4%)	44 (74.6%)	
TAP (μm^2^)				**0.01**
<174	40	17 (42.5%)	23 (57.5%)	
≥174	88	14 (15.9%)	74 (84.1%)	
Tumor location				0.514
Upper	16	11 (68.8%)	5 (31.3%)	
Middle	60	16 (19.2%)	44 (80.8%)	
Lower	52	10 (24.2%)	42 (75.8%)	
Differentiation				**<0.01**
Poor	26	1 (3.8%)	25 (96.2%)	
Well	18	0 (0.0%)	18 (100.0%)	
Moderate	84	30 (35.7%)	54 (64.3%)	

a Chi-square test.

**Table 4:** Characteristics and MPR.

	Case	MPR	No MPR	*p* ^a^
Age (year)				0.836
≤64	60	21 (35.0%)	39 (65.0%)	
>64	68	25 (36.8%)	43 (63.2%)	
Gender				**0.019**
Male	96	29 (30.2%)	67 (69.8%)	
Female	32	17 (53.1%)	15 (46.9%)	
Tumor size (cm)				0.495
≤2.9	70	27 (38.6%)	43 (61.4%)	
>2.9	58	19 (32.8%)	39 (67.2%)	
T stage				0.778
T1–2	18	7 (38.9%)	11 (61.1%)	
T3–4	110	39 (35.5%)	71 (64.5%)	
N stage				0.541
N0–1	49	23 (33.3%)	46 (66.7%)	
N2–3	59	21 (41.2%)	30 (58.8%)	
TAP (μm^2^)				**0.08**
<174	40	21 (52.5%)	19 (47.5%)	
≥174	88	25 (28.4%)	63 (71.6%)	
Tumor location				0.591
Upper	16	16 (37.5%)	10 (62.5%)	
Middle	60	24 (40.0%)	36 (60.0%)	
Lower	52	16 (30.8%)	36 (69.2%)	
Differentiation				**<0.01**
Poor	26	2 (7.7%)	24 (92.3%)	
Well	18	0 (0.0%)	18 (100.0%)	
Moderate	84	44 (52.4%)	40 (47.6%)	

a Chi-square test.

### Survival outcome

**Figure 2:**
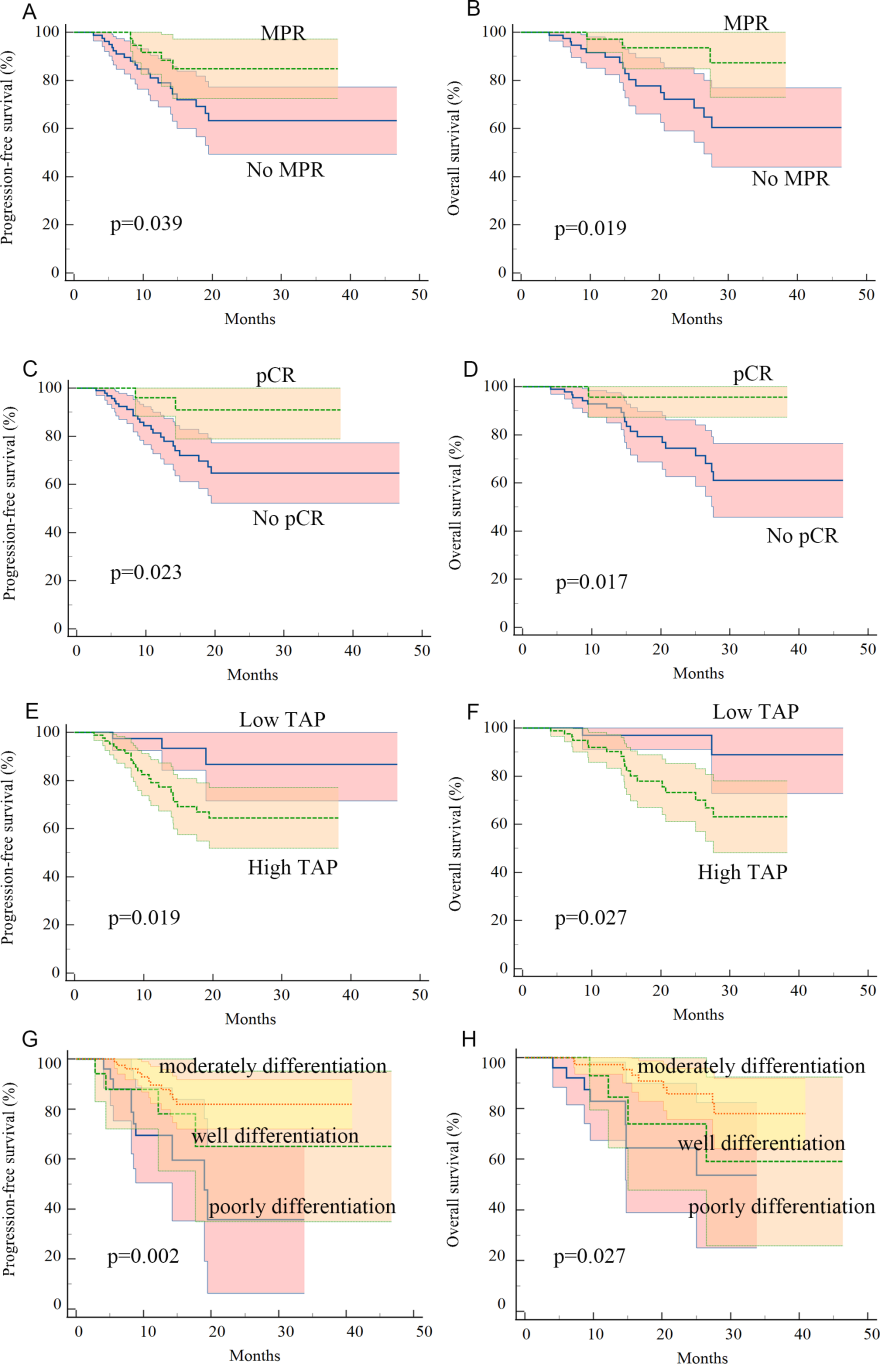
Kaplan–Meier curves for progression-free survival and overall survival in patients with advanced ESCC who received neoadjuvant chemotherapy plus anti-PD-1 immunotherapy: **(A, B)** patients with MPR pathology response had better PFS and OS, **(C, D)** patients with pCR pathology response had better PFS and OS, **(E, F)** patients with low-level TAP before treatment had better PFS and OS, and **(G, H)** patients with moderate tumor differentiation had better PFS and OS.

**Figure 3:**
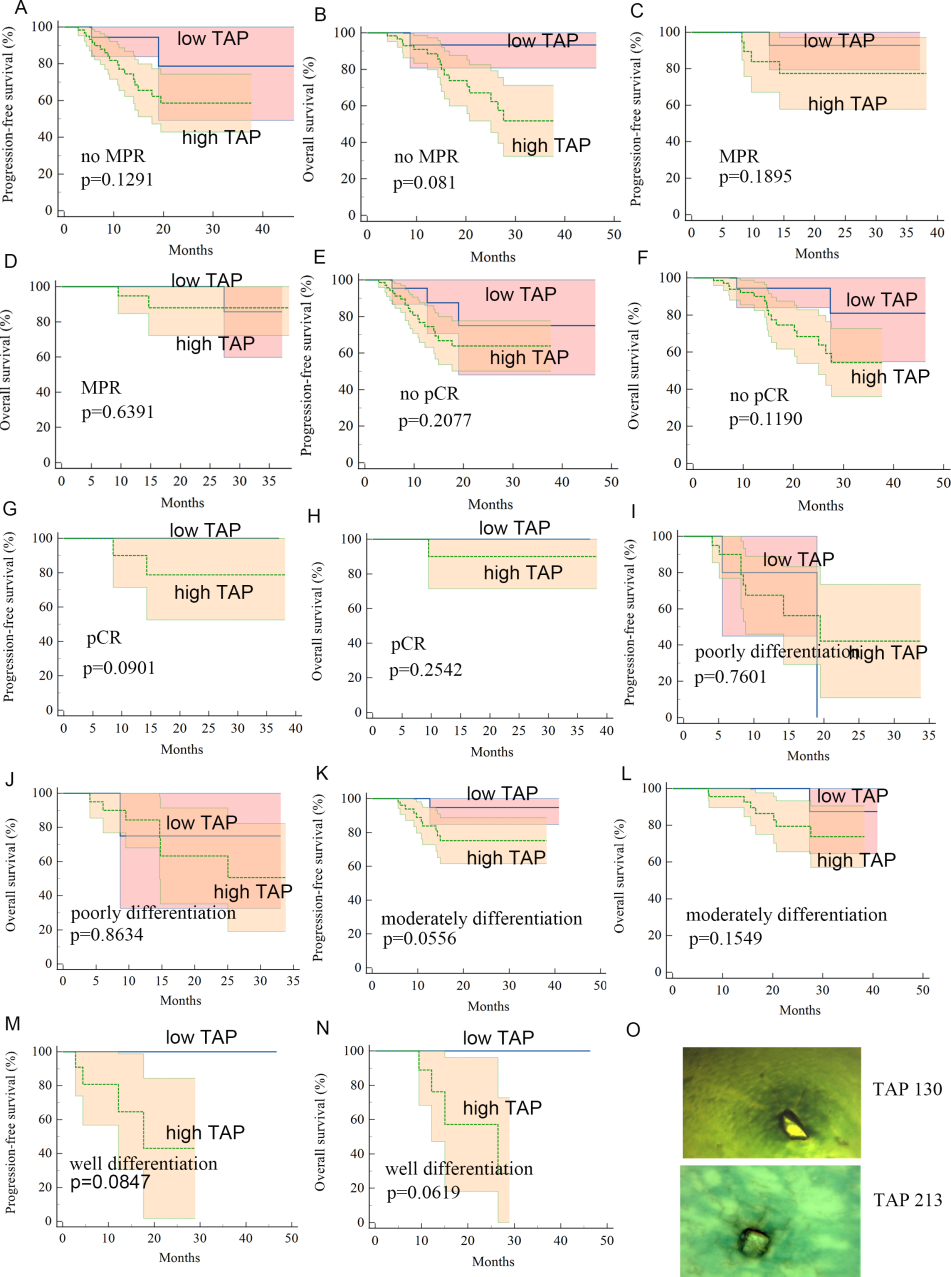
**(A–N)** Kaplan–Meier curves for subgroup patients with low-level TAP or high-level TAP. **(O)** Image of TAP of different values.

**Figure 4:**
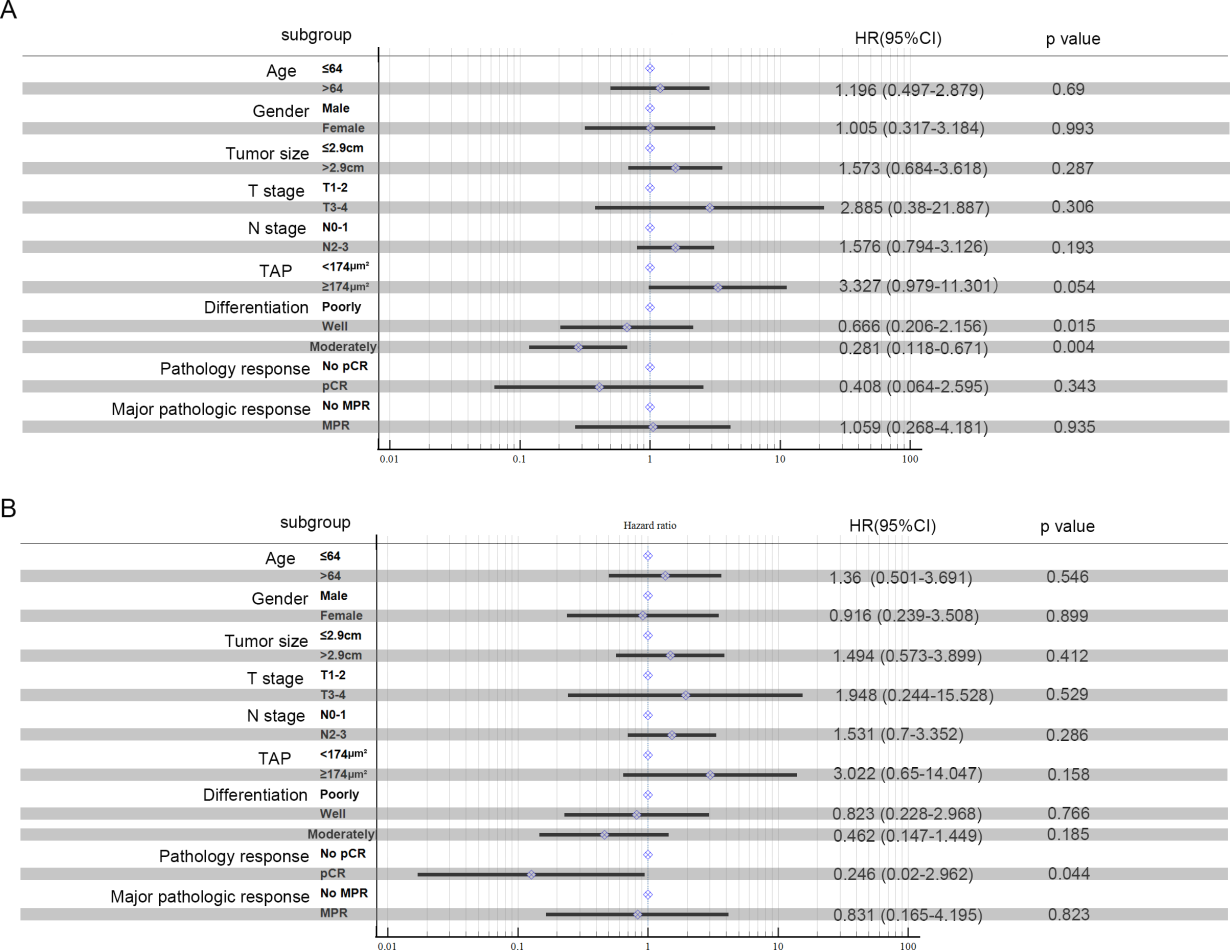
Multivariate analysis: PFS **(A)** and OS **(B)** in ESCC patients treated with neoadjuvant chemotherapy and anti-PD-1 immunotherapy were analyzed.

Kaplan–Meier survival analysis identified TAP levels, tumor differentiation, and pCR and MPR as significant risk factors for progression-free survival (PFS) and OS (**Tables 5**, **6**, **Figures 2**, **3**). Patients with low TAP levels had a mean PFS of 42.4 months (*p* = 0.019) and a mean OS of 43.7 months (*p* = 0.027). Tumor differentiation significantly impacted survival outcomes: patients with moderately differentiated tumors had a mean survival of 35.5 months (HR: 2.146, 95% CI: 0.683–6.742, *p* = 0.002), whereas those with poorly differentiated tumors had the lowest survival, averaging 20.2 months. Furthermore, patients achieving pCR or MPR responses generally experienced improved survival outcomes. In multivariate analysis, high-level TAP was found to be associated with poor PFS (HR: 3.327, 95% CI: 0.979–11.301, *p* = 0.054). The multivariate analysis showed that moderate differentiation was significantly correlated with better PFS (HR: 0.281, 95% CI: 0.118–0.671, *p* = 0.015) (**Figure 4A**), while only pCR rate was found to be significantly associated with good OS (HR: 0.246, 95% CI: 0.02–2.962, *p* = 0.044) (**Figure 4B**). Detailed data are shown in **Tables 5**, **6** and **Figure 4**.

**Table 5:** Characteristics and PFS.

	Case	Mean time	Univariate analysis	*P*
			HR (95% CI)	
Age (year)				0.972
≤64	60	36.558	—	
>64	68	30.288	0.986 (0.450–2.163)	
Gender				0.405
Male	96	35.897	—	
Female	32	31.447	0.672 (0.2646–1.710)	
Tumor size (cm)				0.290
≤2.9	70	31.451	—	
>2.9	58	34.831	1.527 (0.693–3.365)	
T stage				0.181
T1–2	18	35.129	—	
T3–4	110	35.674	3.585 (0.485–26.517)	
N stage				0.466
N0–1	69	37.448	—	
N2–3	59	29.384	1.341 (0.608–2.956)	
TAP (μm^2^)				**0.019**
<174	40	42.413	—	
≥174	88	28.452	3.813 (1.141–12.743)	
Differentiation				**0.002**
Poor	26	20.215	—	
Well	18	34.315	0.524 (0.118–2.328)	
Moderate	84	35.480	0.245 (0.0829–0.724)	
Pathology response				**0.023**
No pCR	97	34.249	—	
pCR	31	35.774	0.218 (0.051–927)	
Major pathologic response				**0.039**
No MPR	82	33.803	—	
MPR	46	34.033	0.371 (0.139–0.990)	

CI, confidence interval; HR, hazard ratio.

**Table 6:** Characteristics and OS.

	Case	Mean time	Univariate analysis	*P*
			HR (95% CI)	
Age (year)				0.765
≤64	60	38.15	—	
>64	68	31.70	1.143 (0.474–2.760)	
Gender				0.469
Male	96	37.47	—	
Female	32	33.50	0.674 (0.232–1.956)	
Tumor size (cm)				0.443
≤2.9	70	32.707	—	
>2.9	58	36.754	1.412 (0.585–3.408)	
T stage				0.293
T1–2	18	35.062	—	
T3–4	110	37.185	2.813 (0.376–21.062)	
N stage				0.828
N0–1	69	38.154	—	
N2–3	59	31.496	1.103 (0.456–2.669)	
TAP (μm^2^)				**0.027**
<174	40	43.689	—	
≥174	88	30.479	4.491 (1.041–19.37)	
Differentiation				**0.027**
Poor	26	24.77	—	
Well	18	34.59	0.682 (0.133–3.477)	
Moderate	84	36.25	0.288 (0.0854–0.973)	
Pathology response				**0.017**
No pCR	97	35.403	—	
pCR	31	37.049	0.127 (.017–950)	
Major pathologic response				**0.019**
No MPR	82	**34.929**	—	
MPR	46	35.946	0.256 (0.075–0.874)	

CI, confidence interval; HR, hazard ratio.

## Discussion

This study examines the effects and prognostic factors in patients with locally advanced ESCC treated with neoadjuvant chemotherapy and anti-PD-1 immunotherapy, followed by surgery at 4–6 weeks later. We identified gender, tumor differentiation, and tumor TAP levels as significant predictors of pCR and MPR rates. Patients with low TAP levels, moderate tumor differentiation, and surgical pCR/MPR exhibited longer PFS and OS.

In Western countries and China, the primary treatment for ESCC is neoadjuvant chemoradiotherapy combined with surgery, while in Japan, neoadjuvant chemotherapy followed by surgery is the standard. Neoadjuvant therapy is thus established as a standard approach for locally advanced esophageal cancer (2, 8). Although neoadjuvant chemoradiotherapy can cause significant adverse effects, immunotherapy has shown considerable efficacy and safety in treating advanced esophageal cancer. Consequently, combining neoadjuvant immunotherapy with chemotherapy for resectable esophageal squamous carcinoma has become a focus of clinical trials, yielding promising results (6, 12, 14, 25, 26). The phase I trials of neoadjuvant immunotherapy have demonstrated substantial efficacy and a favorable safety profile for locally advanced esophageal cancer, enhancing treatment options. However, few studies have explored the prognosis of patients with ESCC treated with neoadjuvant chemoradiotherapy and anti-PD-1 immunotherapy followed by surgery. In our study, the mean OS was 38.0 months (95% CI: 34.8–41.2 months), and the mean PFS was 36.7 months (95% CI: 33.2–40.1 months).

In this study, 96 patients (75%) were male. This gender disparity is due to the main factor: the higher incidence of ESCC in men compared to women (1). Despite this disparity, female patients tend to have better surgical outcomes, suggesting that they benefit more from neoadjuvant chemotherapy combined with anti-PD-1 immunotherapy followed by surgery. Female patients tended to have a stronger immune response than male patients. Sexual hormones can affect lymphocyte infiltration, and sex-related immune factors may promote tumor progression and development more in men than in women (27). Androgen-activated androgen receptors upregulate USP18 expression, which suppresses TAK1 phosphorylation and subsequent NF-κB activation in antitumor T cells. Reduced testosterone synthesis significantly enhances T-cell antitumor activity and improves the efficacy of anti-PD-1 immunotherapy (28). Further investigation is required to elucidate the relationship between sexual hormones and immunotherapy in ESCC.

The goal of preoperative neoadjuvant therapy is to shrink tumors, eliminate or reduce metastases, increase complete surgical resection rates, and improve survival (29). A meta-analysis of 27 clinical trials involving 815 patients found that preoperative immunotherapy combined with chemotherapy in resectable ESCC achieved a disease control rate (DCR) of 99.2% and a pCR rate of 21.9% (30). Clinical trials of neoadjuvant immunotherapy with chemotherapy in advanced ESCC also showed promising pCR rates, including pembrolizumab (7) (41.4%–55.6%), nivolumab (31) (16.7%–59%), camrelizumab (25, 32) (25%–45.4%), sintilimab (33) (21.7%–35.3%), toripalimab (34, 35) (16.7%–36%), and tislelizumab (14) (50%). Our study found a pCR rate of 24.21% in advanced ESCC patients treated with neoadjuvant chemotherapy and anti-PD-1 immunotherapy, consistent with previous studies. Female patients had significantly higher pCR rates than male patients (40.6% vs. 18.8%, *p* = 0.012). Gender differences significantly affect ESCC incidence and prognosis; male patients have a higher incidence, but female patients show better treatment responses and outcomes. Previous studies also indicate that female patients benefit more from neoadjuvant therapy in ESCC (36, 37). Tumor differentiation is another factor influencing the efficacy of neoadjuvant chemotherapy and anti-PD-1 immunotherapy. Moderately differentiated tumors had the highest anti-tumor effect, with a 35.7% pCR rate and a 52.4% MPR rate. ESCC is classified based on atypia and keratinization as well differentiated, moderately differentiated, or poorly differentiated. The function of esophageal keratinocytes correlates with sensitivity to chemotherapeutic agents (27). Poorly differentiated tumors are generally more malignant, with faster progression and poorer prognosis. A scRNA-seq in hepatocellular carcinoma (HCC) developed a differentiation-related gene prognostic index (DRGPI) based on HCC differentiation-related genes (HDRGs) to elucidate the immune characteristics and therapeutic benefits of ICI. A low DRGPI score was associated with high CD8 T-cell infiltration and more benefit from ICI therapy (38). In our study, we found that moderately differentiated ESCC showed the best surgical response. The clear relationship between tumor differentiation and immunotherapy in esophageal squamous cell carcinoma is still unknown. How esophageal keratinocytes act in the immunotherapy and the relationship between esophageal keratinocytes and tumor antigens need more exploration. The lack of basic experimental studies has prevented the details of its molecular mechanism from being elucidated. Further study is urgently needed to elucidate these issues.

This study found that TAP levels are associated with surgical response in patients with ESCC. Patients with low TAP levels had a significantly better response, with a pCR rate of 42.5% compared to 15.9% in the high-TAP group (*p* = 0.01) and a MPR rate of 52.5% *versus* 28.4% (*p* = 0.08). TAP levels correlated with tumor size and regression (**Table 1**). TAP is a critical cancer marker found in various tumor cells and is detectable in peripheral blood. In breast cancer patients receiving neoadjuvant chemotherapy after breast-conserving surgery, TAP levels significantly decreased in those with favorable long-term prognoses. When combined with CA724, TAP showed sensitivity, specificity, and accuracy of 86.00%, 65.22%, and 69.88%, respectively, in evaluating chemotherapy efficacy in gastric cancer patients. TAP combined with CA199 aids in the early diagnosis of biliary tract malignancies. Tumor abnormal proteins are glycosylated glycoproteins with abnormal glycochains. In malignancy, abnormal enzymes on the cell surface alter the sugar chain structure, which can be identified by detecting these proteins. Studying the abnormal glycochain proteins excreted by tumor cells provides a more effective basis for tumor diagnosis. TAP detection technology can simultaneously identify multiple tumor markers, such as AFP and CEA, as well as novel markers, enhancing cancer detection accuracy and reducing missed detections in screenings.

TAP is an abnormal product of tumor metabolism, which is similar to how AFP may be a biomarker; it may be a passenger product of tumorigenesis, and the sexual hormone as basis for individual differences naturally exists in the prerequisite of the tumor. Sexual hormones may drive tumorigenesis and determine the degree of tumor differentiation and then affect the effect of neoadjuvant chemotherapy combined with immunotherapy for esophageal cancer.

Our study has limitations. First, it is retrospective with a small patient sample size. Additionally, the cases span a large timeframe, including the COVID-19 pandemic, affecting data consistency. Most cases require updated follow-up data for a more accurate analysis.

In conclusion, our study found that female ESCC patients treated with neoadjuvant chemotherapy and anti-PD-1 immunotherapy have a significantly higher pCR rate than male patients. Pathological differentiation before neoadjuvant treatment indicates that moderately differentiated tumors respond better than poorly or highly differentiated ones. Pre-treatment TAP plasma levels can serve as predictive indicators for advanced ESCC patients undergoing neoadjuvant chemotherapy and anti-PD-1 immunotherapy.

## Data Availability

The raw data supporting the conclusions of this article will be made available by the authors, without undue reservation.
